# Spinal Cord Stroke: Acute Imaging and Intervention

**DOI:** 10.1155/2012/706780

**Published:** 2012-06-13

**Authors:** Karen Lynch, Joel Oster, Diana Apetauerova, Kinan Hreib

**Affiliations:** ^1^Department of Neurology, Tufts Medical Center, 800 Washington Street, Boston, MA 02111, USA; ^2^Department of Neurology, Lahey Clinic Medical Center, A Tufts University Affiliated Hospital, 41 Mall Road, Burlington, MA 01805, USA

## Abstract

Spinal cord infarction is an uncommon disease and as such is often a diagnostic challenge for clinicians. It can vary in its onset, severity, outcome, and recovery from patient to patient. Treatment options for this relatively rare condition also remain elusive. Current consensus recommendations are antiplatelet therapy and the symptomatic management of associated complications such as paraplegia and thromboembolic disease. There are multiple studies in surgical literature of a variety of interventions and adjuncts used for reducing the risk of ischemic spinal cord neurological injury, seen most often in the setting of thoracoabdominal aortic repair operations. We report two cases of acute non-surgical-related spinal cord infarcts, where early diagnosis was made and aggressive, early treatments instituted. With often devastating outcomes, we highlight the need for early detection and that interventions, commonly used in preventing neurological injury after high-risk aneurysm repairs, may be beneficial in treating and reducing the severity of disability in acute spinal cord stroke.

## 1. Introduction

Spinal cord infarction is an infrequent disease, varying in its presentation, severity, and outcome from patient to patient. This makes it often a diagnostic challenge for clinicians, and with a paucity of interventions and treatments, this serves to add to the task that neurologists face in managing these difficult cases. Reviewing two particular cases in our institution where early diagnosis and aggressive treatments were made, we highlight the possible benefit that could be seen from implementing interventions seen typically in surgical settings for reducing risk of spinal cord ischemia as seen in thoracoabdominal aortic repair operations. 

## 2. Case  1

**Figure 1:**
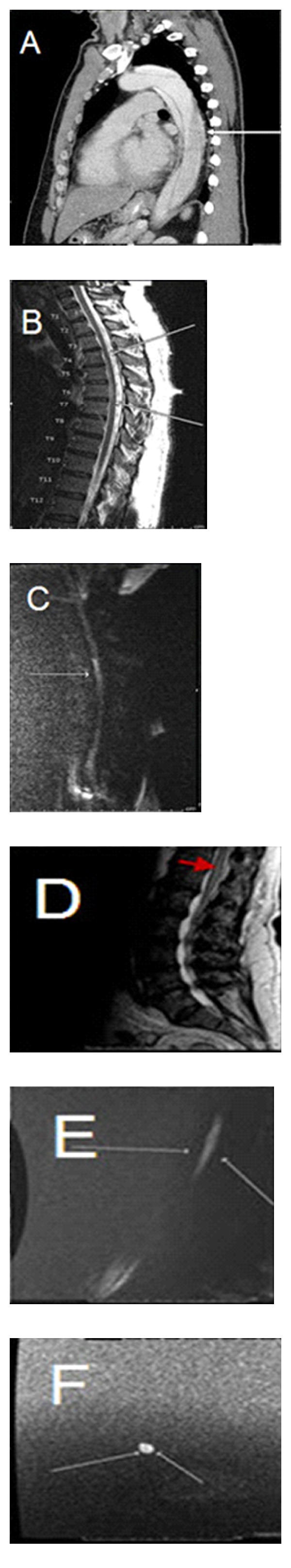
(A) Sagittal CT thorax/abdomen with contrast showing type B aortic dissection beginning distal to the origin of the left subclavian artery. Note the false lumen extending to the inferior mesenteric artery. (B) and (C) Sagittal T2 and DWI MRI of the thoracic spine. Note the abnormal hyperintense signal within the anterior portion of the central gray matter from level T4 to T7 with associated mild cord swelling denoted in (B) and the corresponding diffusion restriction in the same area in (C). (D) Sagittal T2-weighted MRI denoting swelling of the conus with T2 bright signal (red arrow). A sagittal DWI-positive image (E) followed by an axial DWI-positive sequence in (F).

A 54-year-old hypertensive man presented acutely with severe chest and abdominal pain. CT scan of the thorax and abdomen revealed a Stanford Type B aortic dissection (Figure 1(A)). He was admitted to the intensive care unit (ICU) for aggressive blood pressure control and conservative medical management of the dissection. Day 2 of admission, he noticed progressive weakness in both legs and had an episode of bowel incontinence. Neurologic examination was notable for proximal bilateral lower extremity muscle weakness, medical research council (MRC) grade 2/5 at the iliopsoas muscles, with full strength in the remaining muscle groups distally. There was a sensory level to fine touch, pin prick, and temperature from T5-L3 and decreased anal tone with normal perineal sensation on rectal examination. Vibration and proprioception were intact. He was hyporeflexic in the lower extremities with flexor plantar responses bilaterally. 

Magnetic resonance imaging (MRI) of the thoracic and lumbar spine was performed (Figures 1(B) and 1(C)) confirming the clinical suspicion of an acute anterior spinal artery infarct, with likely etiology that of hypotension and decreased perfusion secondary to aortic dissection and concomitant strict blood pressure control. Emergently a lumbar drain was placed to drain cerebrospinal fluid (CSF) in anticipation of possible further cord swelling and edema. CSF pressure was maintained at no greater than 10 mmHg. Scheduled intravenous dexamethasone was also given, and mean arterial pressure (MAP) goals adjusted to at least >70 mmHg (ideally >80 mmHg) to increase perfusion to the spinal cord. 1 week later the patient was able to ambulate with the use of a cane and continued to make an excellent recovery with rehabilitation. 

## 3. Case  2 

A 77-year-old woman with end-stage hypertensive renal disease on hemodialysis presented with an acute sigmoid colon obstruction for which she underwent uncomplicated endoscopic stenting. 12 hours after stenting the patient was noted to have MAP consistently below 70 mmHg, a fever of >101 F, and was delirious. She was treated for possible sepsis, and antibiotics and intravenous fluids were commenced. 8 hours later when more alert, the patient found she was unable to move her legs. Neurological examination was notable for MRC grade 0/5 at all muscles in the lower extremities bilaterally. A sensory level to fine touch, pin prick, and temperature extended up to T10. She was areflexic in the lower extremities with negative Babinski testing bilaterally.

MRI of the thoracic and lumbar spine showed restricted diffusion extending from the lower thoracic cord to the conus (Figures 1(D)–1(F)) confirming a massive acute spinal cord infarct. A CT of her thorax and abdomen ruled out any abdominal aortic dissection, stent migration, or erosion. Further workup for possible differential diagnoses that could explain her imaging abnormalities including infection, inflammation, necrotizing myelitis, and vasculitides was negative. She was transferred to the ICU and commenced on IV dexamethasone with MAP goals of >70 mmHg. Concomitantly she was also found to have an acute non-ST elevation myocardial infarction and started on clopidogrel. Efforts to insert a CSF lumbar drain was not possible due to the chosen antiplatelet and possible bleeding risks of the intervention. The patient was discharged to rehabilitation 2 weeks later when she was medically stable. Neurologically she made a poor recovery, regaining some sensation to a level of L3, but remained paraplegic and incontinent. She died 1 month later of medical complications. 

## 4. Discussion

Spinal cord ischemia is a consequence of multiple complex interactions. Acute hemodynamic instability and malperfusion, oxygen delivery and demand, local metabolic rate, reperfusion injury with intraoperative cases, and a patients' baseline collateral circulation, all play a role in the incidence and severity of spinal cord infarcts. From an anatomical view, three quarters of the blood supply to the cord are derived from the anterior spinal artery, originating from the vertebral arteries, the aorta, and its posterior radicular branches, most notably the artery of Adamkiewicz. Perfusion to the cord can decompensate by occlusion or decreased perfusion of one of these radicular branches or lesions of the aorta, with the midthoracic cord being most vulnerable to watershed infarcts. Thus the anterior portion of the cord is much more vulnerable to vascular compromise, typically presenting as an anterior cord syndrome affecting corticospinal, lateral spinothalamic, and autonomic pathways. Once suspected, confirmation is crucial using diffusion-weighted MRI and prompt initiation of blood pressure augmentation. 

When a diagnosis of spinal cord infarct (SCI) is made, treatment options are extremely limited, with no therapies proven to reverse or limit ischemic spinal cord injury outside the surgical realm. Interventions normally entail augmenting perfusion to an ischemic cord and managing secondary medical complications. Conversely, protocols developed to limit neurologic deficits due to cord ischemia in the setting of aortic or thoracic endovascular repair have been well established in surgical practice [1, 2]. Local hypothermia, steroids, naloxone hydrochloride, mannitol, papaverine hydrochloride, CSF drainage, left heart bypass, barbiturates, and BP augmentation/goals are all used as adjuncts to prevent and treat spinal cord injury in the perioperative and postoperative setting [2]. The perioperative role of CSF drainage is well recognized in thoracic and abdominal aortic surgery with randomized control trials citing up to 80% reduction in the relative risk of postoperative deficits in cases of SCI [1, 3, 4]. Combinations of lumbar drain and intrathecal papaverine have also been successful in reducing the severity of neurological injury. One study also reported CSF drain initiation may reverse cases of delayed onset paraplegia [5]. It must be stressed however that this treatment in spinal cord infarct due to other reasons has not been studied, and with most cases of stroke usually having a delayed diagnosis its benefit would be questionable. 

In our cases, both patients had vulnerability to low perfusion states—renal insufficiency likely an indication of more widespread peripheral atherosclerotic disease, and a relatively low MAP in a chronic hypertensive patient, likely compromised already critical collateral perfusion in both cases. The heterogeneity of the patients and the use of concomitant protective methods make it difficult to evaluate an individual maneuver (contribution of CSF drainage in patient one) as an attribute to subsequent neurological recovery. 

Prompt detection of spinal cord ischemia by neurologic examination and imaging, combined with interventions that increase cord perfusion, is crucial in effectively treating or reversing acute paraplegia or paraparesis. The current standard of care for acute cord infarct is antiplatlet therapy in patients with comorbid atherosclerotic disease; however consideration of instituting some or a combination of therapies used in surgical protocols for preventing spinal cord injury could reverse or limit spinal cord ischemia and minimize the disabling residual neurological deficits that are too frequently seen [6]. Blood pressure augmentation, maintaining higher MAPs, has been proven effective in SCI infarct [7]; however in select patients lumbar CSF drainage should be part of a neurologist's inventory in the management of these difficult cases to further augment spinal cord perfusion pressure and permitting reversibility of SCI and long-term disability. 

## Abbreviations

ICU: Intensive care unit
MRC: Medical Research Council
MRI: Magnetic resonance imaging
CSF: Cerebrospinal fluid
MAP: Mean arterial pressure
SCI: Spinal cord infarct.
